# Controversies in the Staging of Patients with Locally Advanced Cervical Cancer

**DOI:** 10.3390/diagnostics13101747

**Published:** 2023-05-16

**Authors:** Dimitrios Nasioudis, Erin M. George, Janos L. Tanyi

**Affiliations:** Division of Gynecologic Oncology, University of Pennsylvania, Philadephia, PA 19104, USA

**Keywords:** locally advanced cervical cancer, imaging, staging

## Abstract

Approximately 10–25% of patients with locally advanced cervical cancer harbor metastases to the para-aortic lymph nodes. Staging of patients with locally advanced cervical cancer can be performed with imaging techniques, such as PET-CT; however, false negative rates can be as high as 20%, especially for patients with pelvic lymph node metastases. Surgical staging can identify patients with microscopic lymph nodes metastases and aid in accurate treatment planning with the administration of extended-field radiation therapy. Data from retrospective studies investigating the impact of para-aortic lymphadenectomy on the oncological outcomes of patients with locally advanced cervical cancer are mixed, while data from randomized controlled trials do not demonstrate a progression-free survival benefit. In the present review, we explore controversies in the staging of patients with locally advanced cervical cancer and summarize the available literature.

## 1. Introduction

Cervical cancer is the third most prevalent gynecologic malignancy in the United States, with an overall decreasing incidence given the increasing rates of HPV vaccination [1]. However, many patients present with locally advanced disease and require definitive chemoradiation as the standard of care treatment [2]. Approximately 10–25% of these patients will harbor metastases to the para-aortic lymph nodes [3,4,5] (from 11% for patients with IB3 disease to 36% for those with stage IVA disease), which has been identified as an important prognostic factor and can also aid in radiation field planning. Isolated para-aortic lymph node metastases can also be present in up to 25% of patients with locally advanced cervical cancer [3]. With the evolution of imaging techniques, PET-CT can identify approximately 82 to 95% of patients with para-aortic lymph node metastases [3,4,5]. However, the false negative rate can be as high as 22%, especially for patients with positive pelvic lymph nodes [3,4,5]. Thus, several authors have advocated in favor of surgical staging that permits the microscopic examination of lymph nodes and accurate treatment planning with the delineation of radiation fields [4,5]. However, para-aortic lymphadenectomy may be associated with postoperative complications that could delay definitive radiotherapy administration, with increased incidence of lower extremity lymphedema [5]. Currently, there is significant variation in the practice between different centers and providers. In the present review, we summarize the available evidence on the modern clinical staging of locally advanced cervical cancer and discuss the role of surgical staging. 

## 2. Imaging Techniques for Clinical Staging of Locally Advanced Cervical Cancer 

CT, MRI, and PET-CT can be employed for the radiological staging of patients with locally advanced cervical cancer to evaluate the extent of the parametrial spread and bladder or rectal involvement, as well as the presence of lymph node or distant metastases. MRI has superior diagnostic accuracy in assessing direct tumor spread to neighboring structures [6]. On the other hand, PET-CT appears to have superior sensitivity in detecting lymphatic and distant spread. In the ACRIN6671/GOG0233 prospective trial, which enrolled 153 patients with locally advanced cervical cancer, PET-CT had a high specificity (97.7%) and positive predictive value (79.3%) in detecting distant metastases [7]. Cases of false positive results in PET-CT have been reported in some studies [8]. However, since the threshold of PET-CT to detect abnormal lymph nodes is approximately 5 mm, microscopic lymph node metastases cannot be visualized. A recent meta-analysis evaluated the incidence of upstaging following surgical para-aortic nodal dissection for patients with a negative pre-operative imaging. Based on 1138 patients reported in 12 studies who had a negative PET or PET-CT, the pooled incidence of upstaging was 12% (95% CI: 10–15%) [8]. For patients who had a negative MRI or CT scan (*n* = 354), the rate of upstaging was 11% (95% CI: 8%, 16%). However, for patients who had positive pelvic lymph nodes (but negative para-aortic lymph nodes) on pre-operative PET-CT (*n* = 311), the incidence of upstaging was much higher at approximately 21% (95% CI: 17%, 26%), supporting the investigation of surgical staging in this patient population since the benefits may outweigh the theoretical risks. Interestingly, for that specific subgroup, there was no difference in the rate of upstaging for patients who had para-aortic lymph node dissection to the level of the renal vein (12%) or inferior mesenteric artery (10%), suggesting that the latter may be an acceptable boundary when performing surgical staging [8]. 

## 3. Data from Retrospective Studies on the Role of Surgical Staging

Data comparing surgical to clinical staging in the locally advanced cervical cancer population have been mixed with respect to feasibility, safety, and impact on oncological outcomes. In a recent meta-analysis, Delara et al. reported no difference in oncological outcomes between surgical and clinical staging [9] based on data from five studies that reported on a total of 1112 patients with stage IB2-IVA cervical cancer of whom 754 underwent surgical staging [9]. Based on data from four studies, there were no differences in PFS (HR 1.13, 95% CI: 0.73, 1.74) or OS (HR 1.06, 95% CI: 0.66, 1.69) between surgical and imaging staging groups, although significant heterogeneity was found (I-square: 75%) [9]. Among 132 patients who underwent para-aortic LND, the rate of lymph node metastases was 33% (*n* = 44) [9], while the most cephalad (infra-renal vein or inferior mesenteric artery) border was not reported in the majority of studies. 

In a pooled analysis of 685 patients with locally advanced cervical cancer who participated in three large phase III trials (GOG-85, GOG-120, and GOG-165), 555 of them underwent surgical para-aortic lymph node sampling [10]. Surgical staging was optional in one trial, while it was mandatory in the other two. By multivariable analysis radiological staging alone was associated with worse PFS (HR 1.35, 95% CI: 1.01, 1.81) and OS (HR 1.46, 95% CI: 1.08, 1.99), while a higher rate of para-aortic (isolated or in combination with another site) relapse was observed (31.9% vs. 15.1%, *p* = 0.006) in the clinical staging group [10]. The benefit of surgical staging was more evident for patients with advanced-stage disease. However, it should be noted that the utilization of PET-CT during patient enrollment was not widespread; thus, the majority of patients may have been staged with less sensitive imaging modalities, such as CT or MRI. 

In another recent retrospective propensity score matching analysis that included patients diagnosed between 2000 and 2017 from three Mayo Clinic sites, a total of 35 patients with stage IB2-IIIB cervical cancer who underwent surgical staging were matched to 70 patients who had imaging staging alone. A minimally invasive approach was utilized for the majority of the patients (83%), while perioperative complications were noted in three patients. Following a median follow-up of 41 and 51.5 months, respectively, the 5-year PFS (62.6% vs. 72.4%, *p* = 0.77) and OS (70.2% vs. 70.5%, *p* = 0.96) rates were comparable between the two groups, while there were no differences in the incidence of locoregional and distant metastases or long-term complications. However, the median time to radiation therapy initiation was longer in the surgical staging group (median: 47 vs. 28 days), while 31.4% of the patients had modification of their radiation treatment plan based on the pathological results of the para-aortic LND [11]. 

A recent large retrospective study from 10 French oncological centers included 647 patients with locally advanced cervical cancer diagnosed between 1996 and 2016 who received definitive chemoradiation and had no evidence of para-aortic lymph node metastases on their pretreatment CT or PET-CT [12]. Approximately 57% of the patients had PET-CT to assess the lymph node status, while the majority had large tumors (>4 cm) and only 53% received brachytherapy. Surgical staging was performed in 377 (58.3%) of the patients and included the systematic removal of lymph nodes from the iliac bifurcation to the level of the left renal vein. The rates of peri-operative and postoperative complications were 4.8% (mostly vascular injuries) and 13.3%, respectively, while lymph node metastases were detected in 47 (12.5%) patients and resulted in the modification of the radiation therapy fields. Following a median follow-up of 38.1 months, after controlling for confounders, patients who had surgical staging had better DFS (HR 0.64, 95% CI: 0.46, 0.89) and OS (HR 0.43, 95% CI: 0.27, 0.68) survival, while the patterns of recurrence were comparable between the two groups [12]. Interestingly, among patients who experienced a recurrence, the rates of isolated lymph node recurrence were 7.8% and 9% in the staging and no staging groups. The majority of the patients in both groups experienced a multisite (29.4% staging and 36% no staging groups) or distant recurrence (25.5% staging and 26% no staging groups) followed by local (29.4% staging and 22% no staging groups). 

On the other hand, a recent analysis of the US-based National Cancer Database (NCDB) that included 3540 patients with locally advanced cervical cancer diagnosed between 2010 and 2015 reported a low utilization of surgical staging (9.4%) in this patient population, with an overall decreasing trend (from 15.7% for patients diagnosed in 2010 to 8.9% for those diagnosed in 2015) [13]. Patients undergoing para-aortic lymphadenectomy were younger (median age: 46 vs. 52 years, *p* < 0.001) with fewer comorbidities (8.7% vs. 15.6%, *p* < 0.001) and more likely to receive brachytherapy (76.9% vs. 70.9%, *p* = 0.02). Patients who underwent lymphadenectomy had overall adequate staging, with 65.7% having at least 10 lymph nodes removed [13]. There was no difference in the overall survival between the surgical and radiological staging groups even after controlling for confounders (HR 1.07, 95% CI: 0.88, 1.31), while a higher incidence of lymph node metastases was noted in the former group (27.3% vs. 13.2%, *p* < 0.001). Surgical staging was not associated with an overall survival benefit even when evaluating patients who had extensive para-aortic lymphadenectomy (defined as at least 10 lymph nodes removed) or following stratification by disease stage or following exclusion of patients who did not receive brachytherapy [13]. For patients who underwent surgical staging, those with positive para-aortic lymph nodes had worse overall survival even after controlling for confounders (HR 2.09, 95% CI: 1.41, 3.12), underlying the need for novel treatment options in this patient population, especially following the negative results of the OUTBACK trial. The limitations of that study included a lack of central pathology review and data on tumor relapse or mode of imaging used, while the results of the pre-operative imaging and details on the surgical technique employed were not available. 

## 4. Data from Randomized Controlled Trials on the Role of Surgical Staging

Given the potential bias of retrospective studies, several randomized controlled studies (RCTs) have attempted to investigate whether surgical staging is associated with superior oncological outcomes compared to radiological staging for patients with locally advanced cervical cancer without an increase in morbidity [12,14,15]. The first RCT was conducted in Taiwan and enrolled patients with FIGO stage IIB (with tumor size ≥ 4 cm), III, and IVA cervical cancer [14]. All patients underwent pelvic/abdomen CT or MRI and later were randomized to clinical staging alone or surgical staging with the performance of lymphadenectomy from the level of the common iliac bifurcation to the inferior mesenteric artery. Patients with bulky pelvic lymph nodes (≥3 cm in size) and those with CT-guided biopsy-proven para-aortic lymph node metastases were excluded. Following completion of the staging procedure, patients received definitive radiation treatment with extended-field radiation only in the presence of para-aortic metastases [14]. Of note, concurrent chemoradiotherapy was not the standard of care during the period of patient randomization. A total of 61 patients (29 and 32 patients in the clinical and surgical arm, respectively) were enrolled. The study was terminated early when an interim analysis concluded that patients who underwent surgical staging had worse PFS (HR 3.13, 95% CI: 1.42, 6.89) after a median follow-up of 58 months [14]. As expected, the surgical staging arm had a longer time from randomization to radiation initiation (median: 11 vs. 20 days, *p* = 0.001); however, the total treatment time was not statistically different. For patients who had surgical staging, the rate of para-aortic lymph node involvement was 25%, while overall 55% of the patients received concurrent chemoradiation with a slightly higher rate among patients in the clinical arm (65% vs. 47%, *p* = 0.198) [14]. 

Another randomized, controlled trial (LiLACS) was designed to compare pretherapeutic laparoscopic surgical staging followed by tailored chemoradiation to radiological staging alone. The trial was unfortunately terminated early secondary to poor accrual, underlying the difficulty of performing surgical trials in this patient population and the need for international collaborations [15]. Similarly, a pilot phase II study (PALDISC trial) comparing the safety, feasibility, and diagnostic accuracy of PET-CT to surgical staging was cancelled following poor enrollment after only five patients were randomized [16].

The best evidence to date on the role of surgical staging in locally advanced cervical cancer derives from a recently published multi-institutional, international, and randomized control trial (UTERUS-11) that enrolled a total of 255 patients with FIGO 2009 stages IIB-IVA cervical cancer between 2009 and 2013 in 20 centers [17,18]. All patients received guideline-conformant care with pelvic external beam radiation therapy, weekly cisplatin at a dose of 40 mg/m^2^, and brachytherapy. The patients who met the inclusion criteria were randomized 1:1 to radiological staging only (with biopsy in the case of any suspicious imaging findings) and laparoscopic transperitoneal surgical staging that included complete bilateral pelvic and para-aortic lymphadenectomy up to the level of the renal vein [17,18]. The radiological evaluation of the para-aortic lymph node was performed with CT or MRI of the abdomen. PET-CT was permitted but not required and was performed in only 47 patients included in the trial. Extended-field radiation therapy was only administered for patients who had positive para-aortic lymph nodes either pathologically or radiologically, while prophylactic extended-field radiation therapy was not permitted per the study protocol. A total of 121 patients were randomized to the surgical staging arm. No intra-operative death occurred during the surgical staging, while only one (0.8%) patient had conversion to laparotomy, and the incidence of excessive blood loss (>500 mL) was rare (two patients, 1.6%) [17,18]. The overall incidence of any postoperative complication was low (7.3%), demonstrating that laparoscopic surgical staging is safe and associated with minimal peri-operative morbidity [15]. Among patients undergoing surgical staging, 51% had positive pelvic lymph nodes and 24% had positive para-aortic lymph nodes. The overall rate of upstaging was higher in the surgical arm (33%) compared to the clinical arm (8% based on CT-guided lymph node biopsy). Similarly, a higher utilization of extended-field radiation (23% vs. 12%, *p* = 0.02) was noted in the surgical staging arm [17,18]. Interestingly, there was no difference in the time to primary chemoradiation initiation between the two groups (median: 13 vs. 13.5 days from randomization in the surgical and clinical groups, respectively), again supporting that surgical staging in experienced centers is not associated with significant morbidity, which can impact a patient’s ability to receive definitive chemoradiation [17,18]. 

Despite the significant difference in upstaging rates, and the resulting change in radiation therapy treatment planning, there was no difference in disease-free survival between the two groups (aHR 0.73, 95% CI: 0.49, 1.08, *p* = 0.12) [17,18]. However, surgical staging was associated with better cancer-specific survival (HR 0.61, 95% CI: 0.40, 0.93) and in an ad hoc analysis, with better PFS for patients with stage IIB disease (HR 0.51, 95% CI: 0.30 to 0.86) [17,18]. These analyses were not pre-planned and are considered exploratory. Data on patient-reported quality of life and incidence of lymphedema have not been reported. It should be underlined that the majority of relapses in both surgical (87%) and clinical staging (91%) arms involved distant sites, further questioning the impact of para-aortic staging on oncological outcomes. 

As previously discussed, a major criticism of the UTERUS-11 trial was the low utilization of PET-CT. In addition, given the assumption of a relatively high relapse in locally advanced cervical cancer during the study design, the sample size was not optimal. Given the limitations of the aforementioned randomized controlled trials, a new international, multicenter trial (PAROLA, NCT05581121) has been launched and will investigate the impact of para-aortic surgical staging on the 3-year DFS of patients with clinical stage IIIC1 cervical cancer based on pretreatment PET-CT. The study will recruit patients with histologically proven PET/CT stage IIIC1 cervical cancer, and its primary end-point will be disease-free survival. Chemoradiation and brachytherapy will be administered in both arms based on modern techniques, as outlined in the EMBRACE II and ESGO/ESTRO guidelines. In the control arm, patients with at least three pelvic positive lymph nodes detected on PET-CT will also be administered prophylactic para-aortic radiation. In the experimental arm, within 3 weeks from PET-CT, patients will undergo minimally invasive (laparoscopic or robotic-assisted) lymph node dissection that extends from common iliac bifurcation to at least the inferior mesenteric artery. Both extra-peritoneal and intra-peritoneal approaches are permitted. Of note, one ancillary study is SENTI-PAROLA, which is designed to investigate the feasibility and accuracy of para-aortic sentinel lymph node biopsy in this patient population. The PAROLA study is designed as a superiority trial and aims to detect an improvement in 3-year disease-free survival (60% to 70%, HR 0.70). The study aims to recruit 510 patients who will be followed for 5 years after randomization, while an interim analysis for futility and efficacy will be performed following 129 events. Randomization will be stratified based on center, tumor extent (T stage), and receipt of adjuvant treatment [19]. A nonrandomized, multicenter trial (NCT05378087) is currently enrolling patients in China with locally advanced cervical cancer (stages IB3, IIA2, and IIB-IVA) and no radiological evidence of para-aortic lymph node metastases, and based on patient preference, assigns them to either chemoradiation alone or surgical staging followed by chemoradiation [20]. The trial aims to recruit 1956 patients over 5 years [20]. In another randomized phase III trial (CQGOG0103) that started enrollment in January 2020, patients with stage IIICr cervical cancer with radiological findings of bulky lymph node metastases based on CT, MRI, or PET-CT with a short diameter of at least 15 mm are randomized to chemoradiation alone or complete pelvic and para-aortic (to the level of the inferior mesenteric artery) lymphadenectomy followed by chemoradiation [21]. The primary outcome is progression-free survival, while the secondary end-points are 3-year and 5-year OS and surgical complications. The study aims to enroll 452 patients overall for 4 years with a 5-year follow-up. In the clinical staging, only group extended-field radiation therapy will be administered to patients with positive common iliac lymph nodes (short diameter ≥ 10 mm) or positive para-aortic lymph nodes [21] Table 1 summarizes data from randomized controlled trials. 

## 5. Role of Prophylactic Extended-Field Radiation Therapy 

As previously discussed, the aim of clinical and/or surgical staging is the identification of patients harboring para-aortic lymph node metastases and the tailoring of the radiation therapy fields. However, utilization of prophylactic extended-field radiation therapy for patients with radiologically negative lymph nodes but at high risk for occult lymph node metastases based on clinical factors is an alternative strategy that can spare them from the morbidity of surgical lymph node staging [22]. As analyses of the EMBRACE studies have demonstrated, while the overall incidence of nodal failure with modern radiation techniques is relatively low, patients with positive pelvic lymph nodes have higher rates of nodal failure (16% vs. 7%) [23]. Two randomized trials have examined this question, though old radiation techniques were utilized. In RTOG 79-20, which enrolled participants between 1979 and 1986, a total of 367 patients with cervical cancer and either stage IB/IIA with a tumor size > 4 cm or stage IIB were randomized to either standard pelvic radiation or pelvic radiation and para-aortic radiation [24]. The ten-year overall survival rate was higher in the extended-field radiation therapy (55% vs. 44%, *p* = 0.02), though no difference in disease-free survival was observed. However, for patients who had a complete response, those who received the extended radiation therapy arm had lower incidence of distant failures and higher salvage rates of local recurrences. Of note, a higher rate of grade 4 or 5 toxicity was observed in the extended-field radiation arm (8% vs. 4%, *p* = 0.06), though an antiquated 2D technique was used [24]. No benefit was found in another trial that recruited a heterogenous patient population between 1977 and 1981 [25]. However, both trials were conducted in an era before radio-sensitizing chemotherapy was incorporated in the management of locally advanced cervical cancer. The EMBRACE I prospective cohort collected the oncological outcomes of 1416 patients with FIGO (2009) stages IB-IVA cervical cancer who received definitive chemoradiation and image-guided adaptive 3D brachytherapy in 24 specialized centers [26]. While detection of lymph node metastases was performed with a variety of imaging techniques (MRI, PET-CT, and CT scan), all patients received modern radiation techniques. At diagnosis, 48.3% of patients were node negative, 33.7% had positive pelvic lymph nodes, 10.7% had positive common iliac nodes, and 7.5% had positive para-aortic lymph nodes. Following a median follow-up of 34.2 months, the overall rate of nodal failure was 11.4%, with a para-aortic nodal failure rate of 7.8%. By multivariate analysis, elective para-aortic lymph node irradiation was associated with a decreased risk of para-aortic nodal failure (HR 0.53, 95% CI: 0.28, 1.00, *p* = 0.05), while other factors included tumor width as measured by MRI, Hb nadir, and nodal risk group [26]. Based on these results, the protocol of the EMBRACE-II prospective study recommends the administration of prophylactic extended-field radiation therapy (upper border at the level of the renal veins) for patients with at least one abnormal common iliac lymph nodes or those with at least three abnormal pelvic lymph nodes on imaging [27]. A prior retrospective study investigated the oncological outcomes of 198 patients with stages IB2-IVA cervical cancer who had positive pelvic lymph nodes and underwent pelvic radiation therapy with (*n* = 80) or without (*n* = 188) prophylactic extended-field radiation therapy to the level of the left renal vein [28]. Following a median follow-up of 63 months, a lower rate of para-aortic lymph node failure was found among patients who received prophylactic extended-field radiation therapy (1.3% vs. 23.7%, *p* < 0.001). Extended-field radiation therapy was, overall, well tolerated and did not result in increased toxicity [28]. In exploratory analyses, an improved 5-year para-aortic lymph node recurrence-free survival (100% vs. 56.8%, *p* < 0.001) and 5-year cancer-specific survival (93.9% vs. 56.5%, *p* < 0.001) was observed for patients who had positive common iliac pelvic lymph nodes or at least three positive pelvic lymph nodes and not for those who had positive nodes below the common iliac bifurcation or <=2 positive pelvic lymph nodes [28]. The optimal selection criteria for patients who would benefit from prophylactic extended-field radiation therapy are currently under investigation. Other risk factors associated with a high risk of occult para-aortic lymph node metastases include bilateral pelvic lymph node metastases, high levels of squamous cancer cell antigen, and stage IIIB disease. Prediction models have been developed to assist in optimal patient selection; however prospective validation in the context of clinical trials is necessary before widespread adoption [29,30]. Wang et al. used data from 1903 patients and developed a nomogram that takes into consideration histology, tumor size, presence of bilateral pelvic or common iliac lymph node metastases, and pelvic lymph node convergence or muscle involvement [29]. On the other hand, Shim et al. developed a model that incorporates two factors: tumor size and presence of positive para-aortic lymph nodes on PET imaging [30]. Patients with a high-risk score have a 76.2% risk of metastases [30]. Based on a retrospective analysis of 133 patients with stage IIIB cervical cancer, those who received prophylactic extended-field radiation therapy (*n* = 67) had lower incidence of out-of-field recurrence (10.4% vs. 30.3%, *p* = 0.004) and lymph node recurrences (0% vs. 9.1%, *p* = 0.011) [31]. By multivariate analysis, extended-field radiation therapy was associated with improved disease-free survival and out-of-field recurrence-free survival, suggesting a possible benefit for this patient group [31]. In another retrospective study that included 758 patients with locally advanced cervical cancer who did not receive para-aortic radiotherapy, the 5-year para-aortic lymph node recurrence rate was 57% for patients (*n* = 38) with an SCC antigen level >40 ng/mL [32]. However, the benefit of extended-field radiation therapy in this patient population has yet to be demonstrated, especially since high pretreatment SCC antigen levels are associated with a high risk for distant recurrence [33].

As such, based on retrospective data, with modern radiation techniques prophylactic extended-field radiation therapy sterilizes microscopic lymph node involvement and can effectively decrease the risk of para-aortic lymph node recurrence for patients at a high risk for occult para-aortic lymph node metastases with minimal additional morbidity. A recent systematic review of the literature summarized the available evidence and identified studies evaluating the role of extended-field radiation therapy among patients with locally advanced cervical cancer receiving definitive chemoradiation. The authors compiled the available data and identified 11 studies meeting the inclusion criteria [34]. The rate of positive pelvic lymph nodes was 69.6% in the para-aortic radiation group versus 28.6% in the standard radiation group. All but one of the studies excluded patients with suspicious para-aortic lymph nodes. A meta-analysis of survival data from 1113 patients from three separate cohorts demonstrated that prophylactic extended-field radiotherapy (administered in 282 patients) was associated with improved DFS (aHR 0.87, 95% CI: 0.79, 0.97) [24]. The other eight studies did not report a hazard ratio adjusted for confounders. Weighted for study sample size, the average 5-year disease-free survival rate across studies was 72% vs. 67.3% [34]. Para-aortic irradiation reduces the risk of distant metastases for patients with locally advanced cervical cancer, providing the rationale for the use of prophylactic extended-field radiation in patients at risk for occult para-aortic lymph node metastases that can serve as sanctuary sites for distant recurrences [35]. Recent studies utilizing IMRT demonstrated relatively low rates of acute (6–10%) and late (6.5%) grade 3 or higher gastro-intestinal toxicity. Further refinement of the selection criteria with the possible incorporation of molecular biomarkers and radiomics may identify patients at risk for occult para-aortic lymph node metastases. Randomized, controlled trials (KROG 07-01, NCT03955367, NCT 01063387, ChiCTR-IPR-14005499, and ChiCTR-IIR-17013683) are open to enrollment and currently investigating the role of prophylactic extended-field radiation therapy in patients with locally advanced cervical cancer and will provide high-quality data on this practice. 

Delineation of the para-aortic region during radiation therapy delivery is critical to achieving optimal oncological outcomes of para-aortic radiation therapy. Multiple delineation methods for the para-aortic clinical target volume (CTV) have been described in the literature; however, the optimal delineation remains unclear [36,37,38,39,40]. The distribution of positive para-aortic lymph nodes is taken into consideration when designing optimal radiation contouring. In a study that included 72 patients with PET-positive para-aortic lymph nodes, all were located in the inferior third of the para-aortic region, while nodes to the right of the IVC were rare (4%) [39]. Similarly, in another study all positive para-aortic lymph nodes were inferior to the left renal vein, while the incidence of right para-caval nodes was, again, low (5%) [40]. In a retrospective study that included 160 patients with locally advanced cervical cancer who received prophylactic extended-field para-aortic radiation therapy, the para-aortic nodal failure rate was comparable between anatomy-based (*n* = 76) and margin-based (*n* = 84) approaches (1.3% vs. 1.2%). However, the anatomy-based approach was associated with less severe acute gastrointestinal toxicity (13.2% vs. 29.8%, *p* = 0.01) [41]. Improved delineation methods for the para-aortic area when delivering prophylactic extended-field radiation therapy result in significantly decreased radiation exposure of the second and third portion of the duodenum [42].

## 6. Conclusions

For patients with locally advanced cervical cancer, surgical staging can identify patients with microscopic para-aortic lymph node metastases while excluding false positive cases and assisting in accurately delineating radiation fields and avoiding morbidity and long-term complications associated with external beam radiation therapy. When performed in high-volume centers by experienced surgeons, surgical staging is not associated with significant morbidity or a delay in the initiation of definitive chemoradiation. Data from randomized control trials do not support the routine use of surgical staging in this patient population, while data from retrospective studies are inconclusive given the heterogeneity of the studies. With the widespread availability of PET-CT, utilization of prophylactic extended-field radiation, and advancements in radiation therapy techniques, the identification and removal of microscopic lymph node disease may not have a clear impact on oncological outcomes. Given the increased utilization of IMRT, which is associated with decreased toxicity, future trials should also focus on the impact of surgical staging on patients’ quality of life apart from the oncological outcomes.

## Figures and Tables

**Table 1:** Results of randomized controlled trials examining the role of surgical staging in locally advanced cervical cancer.

Study	Surgical Arm	Clinical Staging Arm	Outcome
Lai et al. (2003) [14]	*n* = 32	*n* = 29	Surgical staging a/w worse PFS
UTERUS-11	*n* = 130	*n* = 125	No difference in DFS, surgical staging a/w better CSS
PAROLA	n/a	n/a	Recruiting
LiLACs	n/a	n/a	Terminated
PALDISC	n/a	n/a	Terminated

PFS: progression-free survival; DFS: disease-free survival; CSS: cancer-specific survival.

## Data Availability

Not applicable.
